# First Foods and Gut Microbes

**DOI:** 10.3389/fmicb.2017.00356

**Published:** 2017-03-06

**Authors:** Martin F. Laursen, Martin I. Bahl, Kim F. Michaelsen, Tine R. Licht

**Affiliations:** ^1^National Food Institute, Technical University of DenmarkSøborg, Denmark; ^2^Department of Nutrition, Exercise and Sports, University of CopenhagenFrederiksberg, Denmark

**Keywords:** infant gut microbiota, breast feeding, weaning, gut microbial diversity, complementary diet, transition, SKOT cohorts, family foods

## Abstract

The establishment of the human gut microbiota in early life has been associated with later health and disease. During the 1st months after birth, the microbial composition in the gut is known to be affected by the mode of delivery, use of antibiotics, geographical location and type of feeding (breast/formula). Consequently, the neonatal period and early infancy has attracted much attention. However, after this first period the gut microbial composition continues to develop until the age of 3 years, and these 1st years have been designated “a window of opportunity” for microbial modulation. The beginning and end of this window is currently debated, but it likely coincides with the complementary feeding period, marking the gradual transition from milk-based infant feeding to family diet usually occurring between 6 and 24 months. Furthermore, the ‘first 1000 days,’ i.e., the period from conception until age 2 years, are generally recognized to be of particular importance for the healthy development of children. While dietary changes are known to affect the adult gut microbiota, there is a gap in our knowledge on how the introduction of new dietary components into the diet of infants/young children affects the gut microbiota development. This *perspective* paper summarizes the currently very few studies addressing the effects of complementary diet on gut microbiota, and highlights the recent finding that transition to family foods greatly impacts the development of gut microbial diversity. Further, we discuss potential impacts on child health and the need for further studies on this important topic.

## What Do We Know About Complementary Feeding and Gut Microbiota Development?

The differential effect of breastfeeding as compared with formula feeding or mixed feeding on the composition of the gut microbiota is well established (Penders et al., 2006; Fallani et al., 2010; Bergström et al., 2014; Jost et al., 2015). However, what happens to the gut microbiota when the first solid foods are introduced and gradually replace the milk-based diet has only scarcely been addressed. Longitudinal studies with repeated sampling in a limited number of infants have indicated that microbial composition changes significantly around the period of introduction to solid foods and cessation of breastfeeding/formula feeding (Favier et al., 2002; Wang et al., 2004; Amarri et al., 2006; Roger and McCartney, 2010; Koenig et al., 2011; Thompson et al., 2015). We have previously monitored the fecal microbiota of 330 Danish children participating in the study designated ‘SKOT,’ at age 9, 18, and 36 months. It was evident that *Lactobacillaceae*, *Bifidobacteriaceae, Enterococcaceae* and *Enterobacteriaceae* abundance decreased while *Lachnospiraceae*, *Ruminococcaceae* and *Bacteroidaceae* species increased during the period from 9 to 18 months, i.e., during the period characterized by transition from milk-based feeding to family diet (Bergström et al., 2014). This was largely in agreement with a European study including 531 infants from five different countries demonstrating that consistent compositional changes (decrease in *Bifidobacteriaceae*, *Enterobacteriaceae*, *Clostridiaceae* while increase in *Ruminococcaceae* and *Lachnospiraceae* species) occurred from 6 weeks of age until 4 weeks after the introduction of solid foods, irrespectively of differences in geographic location, use of antibiotics, mode of delivery (vaginal or C-section) and milk feeding practices (Fallani et al., 2011). Recently Thompson et al. (2015) found increased gut microbial diversity of both exclusively breastfed infants (*n* = 4) and mixed-fed infants (*n* = 5) after introduction of solid foods.

**FIGURE 1:**
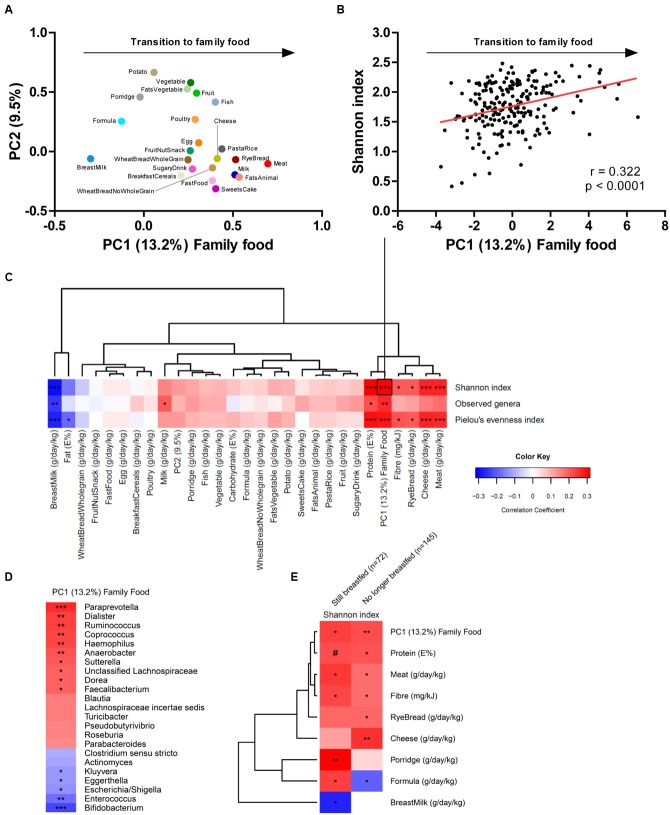
**(A)** PCA loading plot of the 23 food groups (grams per day per kilogram body weight), with the fist principal component defined as family food, describing the transition from early infancy foods to family foods. **(B)** Pearson’s correlation between family food and the Shannon index of alpha diversity. **(C)** Heatmap illustrating hierarchical clustering of correlation coefficients between dietary parameters and alpha diversity metrics. **(D)** Heatmap illustrating correlation coefficients between family food and gut microbial genera showing a *p*-value below 0.1. **(E)** Heatmap illustrating hierarchical clustering of correlation coefficients between dietary parameters and Shannon index in partially breastfed (*n* = 72) and weaned (*n* = 145) infants aged 9 months. Color key applies to panel **(C–E)** and represents correlation coefficients of Spearman’s rank/Pearson’s correlations. Statistical significance is indicated by ^#^*p* = 0.05, ^∗^*p* < 0.05, ^∗∗^*p* < 0.01, ^∗∗∗^*p* < 0.001. The figure is based on previously published data (Laursen et al., 2016).

The general challenge in longitudinal studies is to differentiate between the effects caused by advancing age (leading to increased exposure to environmental microbes and increased maturity of the gut) and effects induced directly by dietary changes. In order to address this issue, we recently combined a comprehensive analysis of the complementary diet with 16S rRNA gene sequence analysis of the fecal microbiota in two independent Danish cohorts of infants aged 9 months, participating in the previously mentioned SKOT study, and demonstrated that gut microbial diversity correlated significantly with the child’s progression toward family foods in both cohorts (Laursen et al., 2016). A Principal Component Analysis of the complete infant dietary records at 9 months, including 23 food groups (recorded in g/day/kg body weight) was performed. The first principal component was defined as “Family Food,” since it described the transition from early infant foods, with high content of breastmilk, formula and porridge (low loadings), to family foods introduced during late infancy (high loadings), with high content of meat, milk, cheese, animal fat and rye bread (type of bread commonly used in complementary feeding in Denmark) (**Figure 1A**). We found separately in both cohorts (Laursen et al., 2016), as well as for the compiled data set (*n* = 217), that the family food parameter was strongly associated with the Shannon index, a commonly used measure of alpha diversity (e.g., within-sample complexity) of the gut microbiota (**Figure 1B**). Other measures of alpha diversity, namely microbial richness (Observed genera) and evenness (Pielou’s evenness index) were strongly associated with the progression to family foods, indicating that both the number of different microbes (richness) and the balance between these (evenness) are affected by this progression (**Figure 1C**). These associations seem to be driven both by the cessation of breastfeeding and by the introduction of food items with higher fiber and protein content, such as rye bread, cheese and meat products, as evidenced from a hierarchical clustering of correlation coefficients between dietary factors and alpha diversity metrics from the compiled data set (**Figure 1C**). Further, progression toward family foods at the of age 9 months was negatively associated with abundances of *Bifidobacterium*, *Enterococcus, Enterobacteriaceae* (*Escherichia*, *Kluyvera*) and to a lesser degree with *Clostridiaceae* (*Clostridium sensu stricto*), but positively associated with abundances of several different bacterial genera (**Figure 1D**), most of them within *Lachnospiraceae* (*Blautia*, *Roseburia*, *Pseudobutyrivibrio*, *Dorea*, *Coprococcus*, *Lachnospiraceae incertae sedis*) and *Ruminococcaceae* (*Faecalibacterium* and *Ruminococcus*), confirming the previous observations from the SKOT cohort (Bergström et al., 2014) as well as other study populations (Fallani et al., 2011; Thompson et al., 2015). Indeed, the relative abundance of species within *Lachnospiraceae* and *Ruminococcaceae* increases when dietary fibers from rye bread (arabinoxylans) or protein from dairy/cheese (casein) and meat are included in the diet (Walton et al., 2012; Zhu et al., 2015). Thus, the introduction of these food components may provide selective advantages for specific microbes to establish in the gut, which will increase alpha diversity. However, we also observed in our cohort studies that the duration of exclusive breastfeeding (range 0–6 months) was more determining for microbial diversity at the age of 9 months than the time of first introduction of solid foods (range 3–6 months), reflecting the dominating impact of breastfeeding (Laursen et al., 2016). To further elucidate the influence of breastfeeding in relation to the effect of complementary diet, we performed a sub-analysis, stratifying the infants by breastfeeding status (defined as at least 1 breastfeeding per day at age 9 months), and correlating the dietary parameters with gut microbial alpha diversity (**Figure 1E**). Protein, fiber and progression toward family foods were positively correlated with the Shannon index in both subsets, indicating that these associations occurred independently of breastfeeding. Interestingly, while meat, cheese and rye bread represented the specific dietary groups most strongly correlated to diversity in the complete dataset (*n* = 217) and in the subset of infants no longer breastfed (*n* = 145), porridge (primarily oatmeal, the most common type of porridge used in complementary feeding in Denmark) was most strongly correlated to diversity in the subset of infants still breastfed at 9 months (*n* = 72). Oatmeal is rich in dietary fibers such as β-glucans, which can be utilized by gut microbes (Hughes et al., 2008). Our results suggest that while infants are still breastfed, porridge is the dietary factor contributing most to gut microbial diversity, whereas when infants are no longer breastfed, consumption of cheese, meat and rye bread is more important. Formula intake was positively associated with diversity in the breastfed infant subset (**Figure 1E**), supporting the previous observations that mixed-fed infants have higher microbial diversity than exclusively breastfed infants (Thompson et al., 2015). Oppositely, formula feeding was negatively associated with diversity in the infants that were no longer breastfed, indicating that the substitution of formula with solid foods such as meat, cheese and rye bread further increase microbial diversity (**Figure 1E**). Together, these results suggest that effects on gut microbial composition and diversity during the complementary feeding period is not solely mediated by weaning/cessation of breastfeeding, but indeed affected by the introduction of new specific complementary foods with higher protein and fiber content.

**FIGURE 2:**
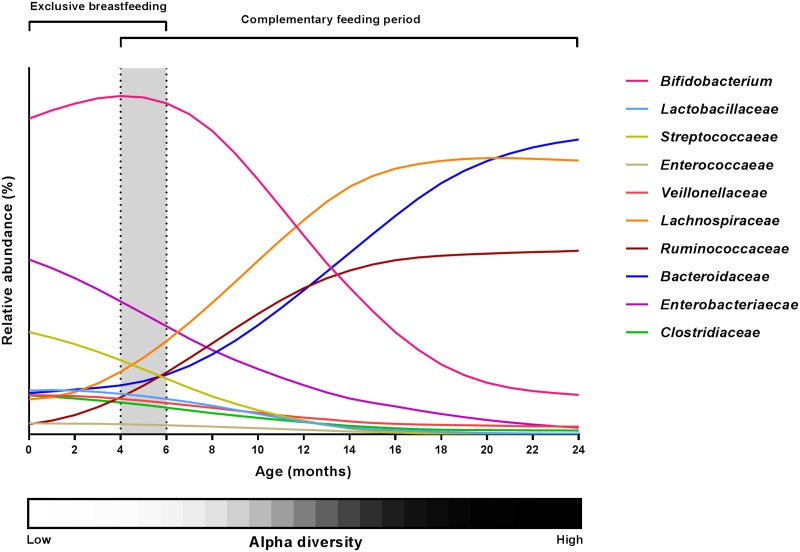
**Changes in the major gut microbial taxa during the first 24 months of life.** Relative abundance estimates and curve shapes are based on average values from available literature (Arrieta et al., 2014; Bäckhed et al., 2015; Kostic et al., 2015; Laursen et al., 2016; Yassour et al., 2016). Individual abundances were normalized to an abundance sum for all bacterial families at each month, and curve smoothing was performed with GraphPad Prism. Dashed lines and gray background indicate the period of introduction to solid foods. Alpha diversity development is shown by a grayscale gradient (white = low diversity, black = high diversity).

The development of the intestinal microbiota is affected by a number of factors including geography, lifestyle and oligosaccharide content of the breastmilk. Although the microbial succession in the infant gut thus greatly varies among individual infants, the general picture emerging (**Figure 2**) is that breast milk keeps the gut microbiota in a state characterized by a high relative abundance of *Bifidobacterium* (with lower abundance of other breast milk associated bacteria such as *Veillonellaceae Enterococcaceae*, *Lactobacillaceae*, *Enterobacteriaceae* and *Streptococcaceae*) accompanied by low diversity, and that these characteristics are affected only to a limited degree by mixed feeding and introduction of the first solid foods as long as the child is still partially breastfed (Roger and McCartney, 2010; Bäckhed et al., 2015; Laursen et al., 2016). However, as complementary feeding progresses, the gut microbial composition changes (increase in the diverse groups of *Lachnospiraceae* and *Ruminococcaceae* species and decrease of *Bifidobacterium*, *Enterobacteriaceae*, *Enterococcaceae, Lactobacillaceae*, *Veillonellaceae, Clostridiaceae*) and total diversity increases (Favier et al., 2002; Roger and McCartney, 2010; Laursen et al., 2016), probably as a result of the increased fiber and protein content of the diet (Laursen et al., 2016). Finally, cessation of breastfeeding/formula feeding and complete transition to family food marks another important event in gut microbial development (Favier et al., 2002; Roger and McCartney, 2010; Bäckhed et al., 2015), characterized by an increased abundance of *Bacteroidaceae, Lachnospiraceae* and *Ruminococcaceae* and a further reduction in *Bifidobacterium* as well as increased alpha diversity (Roger and McCartney, 2010; Bergström et al., 2014; Bäckhed et al., 2015).

## What Does it Matter for Child Health?

The consistently observed increase in gut microbial alpha diversity during the time period coinciding with complementary feeding (Yatsunenko et al., 2012; Kostic et al., 2015; Avershina et al., 2016) and recent evidence showing that this increase is driven by transition from breastfeeding to family foods (Laursen et al., 2016), raise some key questions: Is the low diversity sustained by breastfeeding beneficial for infant health? Does the increase in microbial diversity observed during complementary feeding characterize healthy gut microbiota development?

Many studies have linked low gut microbial diversity to diseases in adulthood as recently reviewed (Mosca et al., 2016). Conditions associated with low gut microbiota diversity range from gastrointestinal pathologies such as Crohn’s disease, ulcerative colitis, irritable bowel syndrome and colorectal cancer to metabolic disorders including obesity, type 2 diabetes, as well as neurological conditions such as autism (Mosca et al., 2016). Recent studies reveal that an important determinant of high microbial diversity in adult fecal samples is a long intestinal transit time (Vandeputte et al., 2015; Falony et al., 2016; Roager et al., 2016). In this context, we have recently proposed that a high microbial richness and diversity may not imply *per se* a healthy gut microbial ecosystem, as longer transit times are significantly associated with increased bacterial protein degradation, which results in potentially detrimental bacterial metabolites (Roager et al., 2016). During the complementary feeding period, transition to family foods implies increased protein and fiber intake, which increases microbial diversity (Laursen et al., 2016). While the implications of this transition on intestinal transit time are not known, bowel movement frequency is generally reported not to change significantly during the weaning period (Tunc et al., 2008).

In infants, the major determinant of a low microbial diversity is probably breast milk (Azad et al., 2013a; Thompson et al., 2015; Laursen et al., 2016), which selects efficiently for specific bacterial species capable of degrading particular oligosaccharides present in breastmilk, the so-called human milk oligosaccharides (HMOs). HMO-degrading species include *B. bifidum, B. longum* ssp. *infantis* and to a lesser degree *B. breve* (Sela and Mills, 2010), while, noteworthy, bifidobacterial species common in the adult gut (e.g., *B. adolescentis*) do not share the capacity to degrade HMOs (Sela and Mills, 2010). *In vitro* studies suggest that the infant-type bifidobacteria play an important role in the maturation of the child’s immune system (Underwood et al., 2014). In line with this, breastfeeding promotes several health effects that are likely to be caused by an improved immune response, such as a reduced incidence of childhood infections (Victora et al., 2016). This suggests that the predomination of infant-type bifidobacteria, implying a low bacterial diversity during breastfeeding, is beneficial for child health. In contrast, some health disorders have been linked to a reduced microbial diversity in early life. Examples include development of eczema (Forno et al., 2008; Wang et al., 2008; Abrahamsson et al., 2012; Ismail et al., 2012) and asthma (Abrahamsson et al., 2014), which have been linked to low microbial diversity in very early life (age 1 week – 4 months). Additionally, in children aged 18–24 months, low microbial diversity has been shown to be a risk factor for development of type 1 diabetes (Giongo et al., 2010; Kostic et al., 2015). Importantly, the low microbial diversity in these studies was not coupled to *Bifidobacterium* abundance and no negative effects of breastfeeding on prevalence of asthma, allergies or development of type 1 diabetes have been reported (Victora et al., 2016). Thus, even though early life low microbial diversity seems to be associated with diseases later in childhood, this is not likely to be caused by *Bifidobacterium* predominance or prolonged breastfeeding. Importantly, the microbial taxa that are absent or otherwise driving low diversity in the above-mentioned studies and their causality to diseases remain to be identified. Some studies suggest that an insufficiently developed microbial community in the gut can be “repaired,” e.g., it has recently been demonstrated that an immature gut microbiota of stunted children aged 6 months, which is within the aforementioned window of opportunity, may at this age be repaired by introduction of adult-like microbes such as *Ruminococcus gnavus* and *Clostridium symbiosum*, both belonging to the *Lachnospiraceae* (Blanton et al., 2016), a microbial family greatly increasing during introduction of family foods (Laursen et al., 2016). The normally observed development across human populations is an increasing microbial diversity with age during the first 3 years of life (Yatsunenko et al., 2012) and probably characterizes a healthy development. While proteins and fibers were found to increase gut microbial diversity (Laursen et al., 2016), a high-protein intake in the complementary feeding period may increase the risk of obesity later in life (Lind et al., 2017), and defining recommendations for fiber intake in early life is equally challenging (Aggett et al., 2003; Zalewski et al., 2015). Consequently, even though gut microbial diversity increases with protein and fiber intake during the complementary feeding period, there is no evidence to support that excessive intake of these macronutrients in early childhood is beneficial for child health.

In summary, we want to emphasize that findings regarding the diversity of the intestinal microbiota in adults cannot be extrapolated to children, since the factors governing the co-development of the microbial ecosystem and the immune system in infanthood are completely different from the factors of importance for adult health. A low diversity of the infant gut microbiota in early life must thus be expected to characterize a healthy gut, if caused by breastfeeding. However, later progression into a diverse gut microbiota during childhood seems to be a fundamental part of the natural succession of the gut ecosystem and characterizes healthy development. One of the main challenges remaining is to identify more accurately the best timing of breastfeeding cessation and of introduction of family foods. This timing is influenced by many other factors than the microbiota, and is likely to be determined by the level of maturation of the infant’s immune system.

## What is Needed to Expand Our Knowledge on Complementary Diet and Gut Microbes?

Until now, only a few studies have performed dietary assessment during the complementary feeding period and compared this with gut microbiota development. To decipher the effects of specific food components on gut microbial development, detailed assessments of the complementary diet constituents are needed. If food frequency questionnaires (FFQ) are used, they should be validated before application to minimize the risk of over/underestimation because they are prone to recall bias. Additionally, it should be considered that FFQs are generally developed to depict long-term or habitual diets, which may not be appropriate during a period of rapid dietary changes. Twenty-four hour dietary recalls are less prone to recall bias, but do not capture day-to-day variations in the diet. The most detailed and accurate results are obtained from food records for periods of 5–7 days since they collect actual intake information from a specific short-term period and are not associated with recall bias (Shim et al., 2014). Validated food records that minimize the risk of under/overestimation were used in our SKOT cohort studies, and because they were conducted during the time period of fecal sample collection, they allowed us to directly assess the impact of the complementary diet on gut microbiota composition (Laursen et al., 2016). Ideally, longitudinal studies should include several diet recordings across the complementary feeding period to capture the individual dynamics during this period of multiple dietary changes.

To capture changes in the gut microbiota, either 16S rRNA amplicon or shot-gun based community sequencing can provide detailed information of the entire microbial community, while shot-gun based sequencing additionally provides direct information on the functional capacity of the bacterial population. However, while these methods generate a large amount of useful data, they lack enough sensitivity for low abundant microbial species. Therefore, complementing these techniques with quantitative PCR approaches and culture-based approaches provide better sensitivity, but are more laborious and time consuming. To minimize confounding effects on the gut microbiota in cross sectional studies, accurate standardization of the age of infants at which the samples are taken is highly desirable. Additionally, it is important to consider adjustments for other factors known to affect infant gut microbiota, including antibiotic intake, C-section, childcare/daycare, geographical location, family environment and exposure to pets and siblings (Yatsunenko et al., 2012; Azad et al., 2013b; Laursen et al., 2015; Thompson et al., 2015; Martin et al., 2016; Yassour et al., 2016). In order to establish causal relationships between specific components of the complementary diet and gut microbial composition, intervention studies are required. This could be done by performing short-term intervention studies, where the effect of specifically designed differences in dietary composition can be tested. Inclusion of the above-mentioned factors in the design of future studies will enable us to expand our knowledge on the impact of the complementary diet on the infant gut microbiota.

## Summarizing Remarks

The very first nutrition most infants receive, breastmilk, keeps the microbiota in a state characterized by low diversity and *Bifidobacterium* domination, which is likely to be beneficial for child health. Introduction of complementary feeding and the transition to family foods increase gut microbial diversity and is accompanied by an increase of adult-associated microbes belonging to the families *Lachnospiraceae* and *Ruminococcaceae*. This increase in diversity occurs across different human populations and is likely to characterize a normal and healthy development of the gut microbiota, since perturbation of the process has been linked to an increased risk of diseases later in life. An important remaining challenge is to determine the optimal age for increasing intestinal microbial diversity by reduction of breastfeeding and introduction of family foods in order to optimize effects on gut microbiota, immune maturation and later health outcomes.

## Ethics Statement

The present perspective uses data from previous publications based on the SKOT cohorts, in which 311 (SKOT I) and 184 (SKOT II) Danish children were followed for the first 3 years after birth, with the overall aim of investigating relationships between early diet, growth development, and later disease risks, especially obesity and metabolic syndrome. The study protocols were approved by the Committees on Biomedical Research Ethics for the Capital Region of Denmark (H-KF-2007-0003 and H-3-2010-122).

## Author Contributions

ML, MB, KM, and TL conceived the idea for this perspective, and all contributed to the outline. All four authors were significantly involved in the studies resulting in the data presented in **Figure 1**. ML drafted the manuscript and the figures. All authors contributed to the writing, and approved the final manuscript.

## Conflict of Interest Statement

The authors declare that the research was conducted in the absence of any commercial or financial relationships that could be construed as a potential conflict of interest.
